# Symptomatic treatment of infantile nystagmus: a systematic review

**DOI:** 10.3389/fnins.2025.1612504

**Published:** 2025-06-19

**Authors:** Xuanwei Li, Bianca Huurneman, Jeroen Goossens

**Affiliations:** ^1^Donders Institute for Brain, Cognition and Behavior, Radboud University, Nijmegen, Netherlands; ^2^Department for Medical Neuroscience, Radboud University Medical Center, Nijmegen, Netherlands; ^3^Royal Dutch Visio, Nijmegen, Netherlands

**Keywords:** infantile nystagmus, symptomatic treatment, perceptual training, visual acuity, methodological heterogeneity

## Abstract

Infantile nystagmus (IN) is a neuro-ophthalmic condition characterized by involuntary rhythmic eye movements that manifest early in childhood. The surge of recent articles focusing on the treatment of IN demonstrates the need for a new systematic review of the intervention options. The diverse causes of IN complicate its differentiation from symptoms secondary to other conditions, presenting challenges for clinical decision-making and systematic review. This study provides the first extensive, focused summary of symptomatic treatment options for IN. We noted that current approaches can be broadly categorized into four types of interventions: surgical, pharmacological, optical, and perceptual training methods, each offering distinct benefits and limitations. Most of the included studies (28/52) focused on invasive surgical interventions. Alternatively, the nascent perceptual training showed promising improvements in both visual acuity (VA) and quality of life (QoL). Heterogeneous reporting of treatment outcomes for IN hindered meta-analysis and precise comparison of intervention effects, underscoring an urgent need for standardized outcome measures in future studies. We further suggest including functional vision measurements and QoL assessments to better address patient well-being, rethinking invasive surgical approaches and exploring non-invasive treatment modalities in clinical practice.

## 1 Introduction

For an object to be perceived with clarity, it is imperative to ensure a steadfast fixation of its image onto the fovea of the retina. Nystagmus, an involuntary rhythmic oscillation of the eyes, can disrupt the fixation and result in blurred vision and illusory motion (Thurtell and Leigh, 2011). The manifestation of these visual symptoms can induce considerable distress and result in substantial disability, impeding various aspects of an individual’s self-esteem, social functioning, and quality of life (QoL) (Pilling et al., 2005; McLean et al., 2012). The sign of nystagmus is rhythmic, regular, and sustained compared to saccadic intrusions (Rucker, 2019). The mechanisms of nystagmus remain incompletely understood. However, based on the waveform characteristics and, primarily, the onset time, nystagmus is categorized into two main types: infantile nystagmus (IN) (previously termed congenital nystagmus) and acquired nystagmus (Papageorgiou et al., 2014).

Typically, IN is present and diagnosed from the age of 3 ~ 6 months onwards while acquired nystagmus appears later (Papageorgiou et al., 2014; Ospina, 2018; Fossataro et al., 2024). It is common for IN to manifest as an isolated symptom, whereas the majority of cases in acquired nystagmus exhibit a confluence of additional indicative factors that point toward the underlying etiology (Ospina, 2018). Acquired nystagmus often arises from lesions affecting the visual feedback loop, the vestibular-ocular reflex circuits, and the neural eye position integrators, while IN is characterized by different but enigmatic etiologies (Rucker, 2019; Leigh et al., 2002). Various hypotheses about the pathophysiological mechanisms of IN have been proposed, including instability within the optokinetic system, dysfunction of the neural integrator, aberrations in the foveal pursuit system, and conflicting interplay between the primitive subcortical optokinetic pathway and cortical smooth pursuit system (Yee et al., 1980; Optican and Zee, 1984; Jacobs and Dell’Osso, 2004; Brodsky and Dell’Osso, 2014). Mutations in *FRMD7*, *TUBB3*, or L-dopachrome tautomerase were demonstrated to cause IN (Tarpey et al., 2006; Jin et al., 2021; Volk et al., 2021). The heterogeneity of the causes adds up to the challenge of distinguishing IN from symptoms secondary to other conditions, complicating the clinical decisions and its systematic overview.

Although the exact pathophysiological underpinnings of IN are yet to be elucidated, clinical treatment is needed, as IN represents the predominant form of nystagmus, accounting for the majority of the cases (58%), particularly in pediatric practice (75–83%) (Sarvananthan et al., 2009; Ehrt, 2012; Hertle et al., 2022). Patients with IN often do not receive treatment, either due to an absence of complaints or the prevailing belief that individuals must adapt to living with the condition (Oogfonds, 2023). For patients in need, treatment options do exist and various academic studies have explored different treatment approaches for IN (Hertle, 2022; Self et al., 2020). However, the available literature summaries in this field are either focusing on acquired nystagmus or nystagmus generally (Thurtell and Leigh, 2010; Tegetmeyer, 2014; Thurtell, 2015; Käsmann-Kellner, 2017; Strupp et al., 2021), limited to a single treatment modality (Zahidi et al., 2017; Cham et al., 2021; Chang et al., 2023), outdated (Abel, 2006; Richards and Wong, 2015), constrained in geographic locations (Self et al., 2020), or written in a non-English language (Pan, 2021). Moreover, almost none of these summaries encompass the significant body of behavioral studies that have demonstrated promising potential in the treatment of IN in recent years.

Before reviewing treatment methods, it is important to highlight key characteristics of IN: Key clinical features include predominant horizontal oscillations, with vertical and torsional oscillations being less common (Ospina, 2018). IN exacerbates during distance fixation and diminishes when the patient engages in near-vision tasks that involve convergence (Rucker, 2019; Ospina, 2018). A null zone, where nystagmus intensity decreases, is common. If offset from the primary position, individuals may adopt an anomalous head posture (AHP) to enhance foveation (Ospina, 2018). This posture commonly involves a head turn or head tilt. IN can occur in association with ocular disease, neurological disorders, or genetic syndromes, though many cases are idiopathic (Self et al., 2020; Käsmann-Kellner, 2017). A Danish study of 103 patients found a 32% prevalence of idiopathic infantile nystagmus (IIN) among children with IN (Hvid et al., 2020).

IN treatments generally include causal and symptomatic approaches. This review focuses on symptomatic treatments, often used for IIN or when causal options are unavailable, as in albinism or optic nerve hypoplasia (ONH) (Lohmüller et al., 2017; Liu et al., 2021). Symptomatic treatments are mostly considered in patients with abnormal gaze, restricted head posture, or decreased visual acuity (VA) (Self et al., 2020; Richards and Wong, 2015). Common evaluation of patients with IN includes nystagmography, gaze and posture, best-corrected visual acuity (BCVA), reading performance, and QoL (Self et al., 2020; Käsmann-Kellner, 2017). Articles published before 2015, including work by Bedell, Dell’Osso, and others, have been extensively reviewed. This updated review focuses on studies since 2015. After pre-screening, we identified four categories of current interventions: surgical, pharmacological, optical, and behavioral. Clinically, refractive correction is always the first step in addressing low vision in IN (Self et al., 2020). Thus, only articles emphasizing optical correction as the primary intervention to reduce nystagmus or improve VA are included in the optical modality section.

## 2 Materials and methods

### 2.1 Systematic literature search

The most recent literature search was conducted on August 8th, 2024. Literature that concerned IN and its symptomatic treatment options between January 2015 and July 2024 were selected, screened, and assessed. The search was conducted by selecting “infantile nystagmus” or “congenital nystagmus” in the titles or Mesh keywords, selecting “treatment,” “intervention,” “therapy,” “approach,” “improvement” or their derived words in titles or abstracts (Table 1). The search was not limited to English.

**Table 1:** Inclusion criteria.

(a) Search history in PubMed.		
Search	Most recent queries	Result
#1	Search ((Infantile nystagmus[Title]) OR (Infantile nystagmus[MeSH Major Topic]) OR (congenital nystagmus[Title]) OR (congenital nystagmus[MeSH Major Topic]))	948
#2	Search ((treatment[Title/Abstract]) OR (treatments[Title/Abstract]) OR (intervention[Title/Abstract]) OR (interventions[Title/Abstract]) OR (therapy[Title/Abstract]) OR (therapies[Title/Abstract]) OR (approach[Title/Abstract]) OR (approaches[Title/Abstract]) OR (improvement[Title/Abstract]) OR (improved[Title/Abstract]) OR (improves[Title/Abstract]) OR (improve[Title/Abstract]))	11,238,579
#3	Search #1 AND #2 AND (2015:2024/07[pdat])	97
(b) PICO—Criteria used for this systematic review.		
P: Population of Interest	Patients with IN / congenital nystagmus	
I: Intervention	Symptomatic treatment of IN, including Surgical intervention Pharmacological therapy Optical correction Perceptual training	
C: Control	Pre-treatment condition	
O: Outcome	Various outcome measurements, including Nystagmography VA QoL	

### 2.2 Study selection

Study titles and abstracts were assessed by author X.L. to determine whether the article should be selected for a full-text review and the process was supervised by author J.G. In cases of doubt, a third reviewer (author B.H.) was consulted.

### 2.3 Inclusion criteria

The following criteria were used for study selection (Table 1):

1. The article should contain or focus on IN instead of acquired nystagmus.
2. The primary objective of the article should be the treatment or intervention of IN rather than the etiology, the discovery of the pathology, or the diagnostic pattern.
3. The treatment option should focus on the symptomatic approach rather than the cause of the primary disease such as certain oculomotor disorders, brain tumors, stroke, or gene mutation.

Given the limited hits, the literature review on optical and pharmaceutical interventions was expanded by sourcing additional records, including citation lists from relevant reviews.

## 3 Results

**Figure 1:**
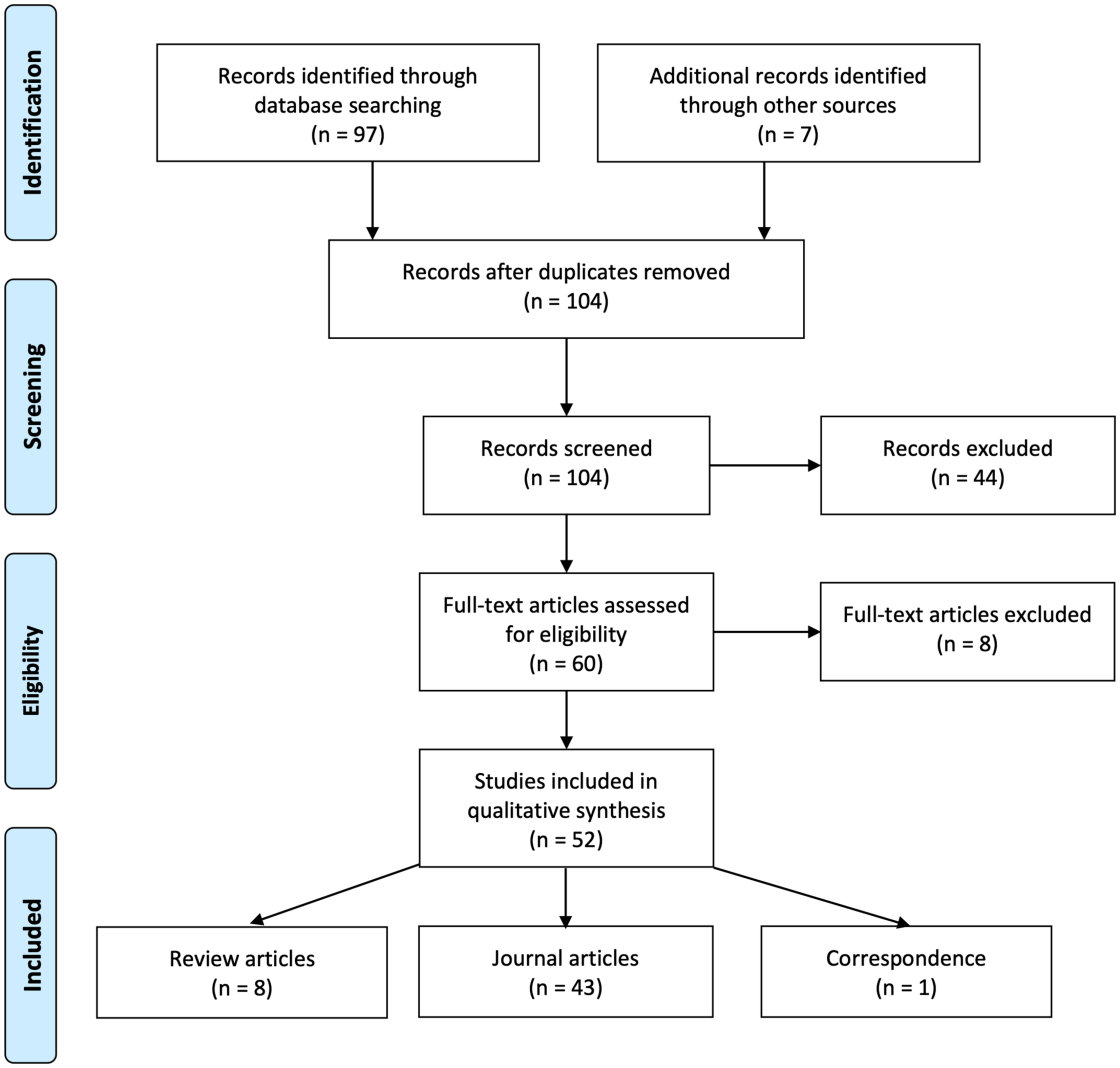
PRISMA flowchart depicting the study identification, screening, eligibility assessment, and inclusion stages of the literature selection process (Moher et al., 2009).

The initial search terms resulted in 97 articles and an additional 7 articles ranging from 2004 to 2011 were included by citation searching. After careful screening, 60 journal articles were selected in the full-text review based on their abstracts according to the inclusion criteria. Of the included articles for full-text review, 8 articles were excluded after examination. The remaining 52 articles comprised 8 review articles, 43 journal articles, and 1 correspondence. The journal articles were categorized by the primary type of intervention. The results were as follows: 23 studies were found on surgical interventions (from 2015 to 2023), 7 covering pharmacological therapy (from 2007 to 2023), 5 studies about optical correction (from 2004 to 2023), 7 studies about behavioral training (from 2015 to 2023), and 1 article with no intervention (2017). For an overview of the selection process, see the PRISMA flow chart (Figure 1). Additionally, this review comprehensively examined 8 comprehensive review articles ranging from 2015 to 2023, with two focusing on surgery for IN (Cham et al., 2021; Chang et al., 2023), one on optometry (Zahidi et al., 2017), two written in German (Tegetmeyer, 2014; Käsmann-Kellner, 2017), and one in Chinese (Pan, 2021), one general review paper on IN (Richards and Wong, 2015), and one broader review on nystagmus (Ospina, 2018).

**Figure 2:**
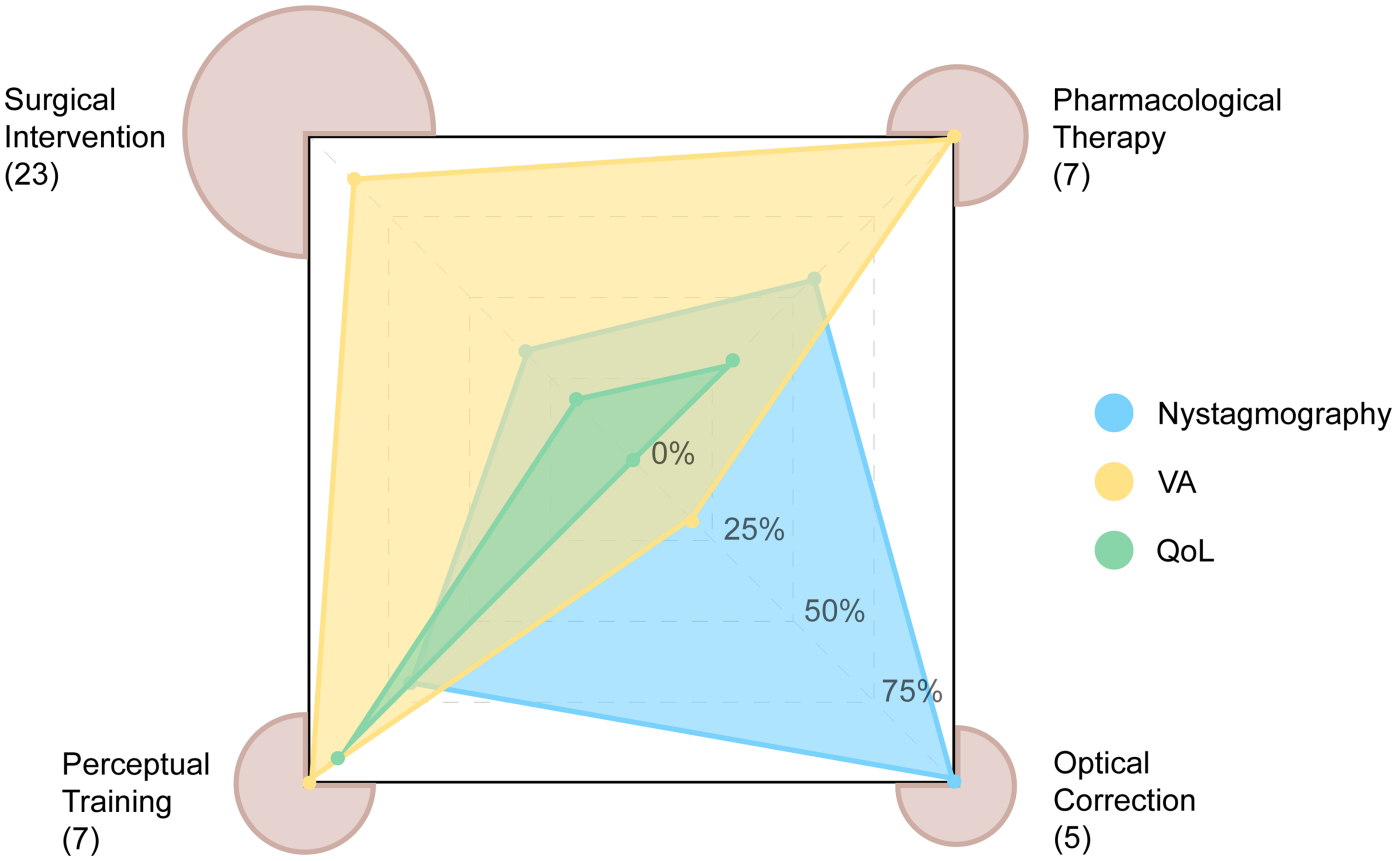
Radar plot showing the number of studies categorized by primary treatment, along with the percentages of reported outcome measures: nystagmography, visual acuity (VA), and vision-related quality of life (QoL). Note the substantial heterogeneity in the reporting of primary outcome measures.

**Figure 3:**
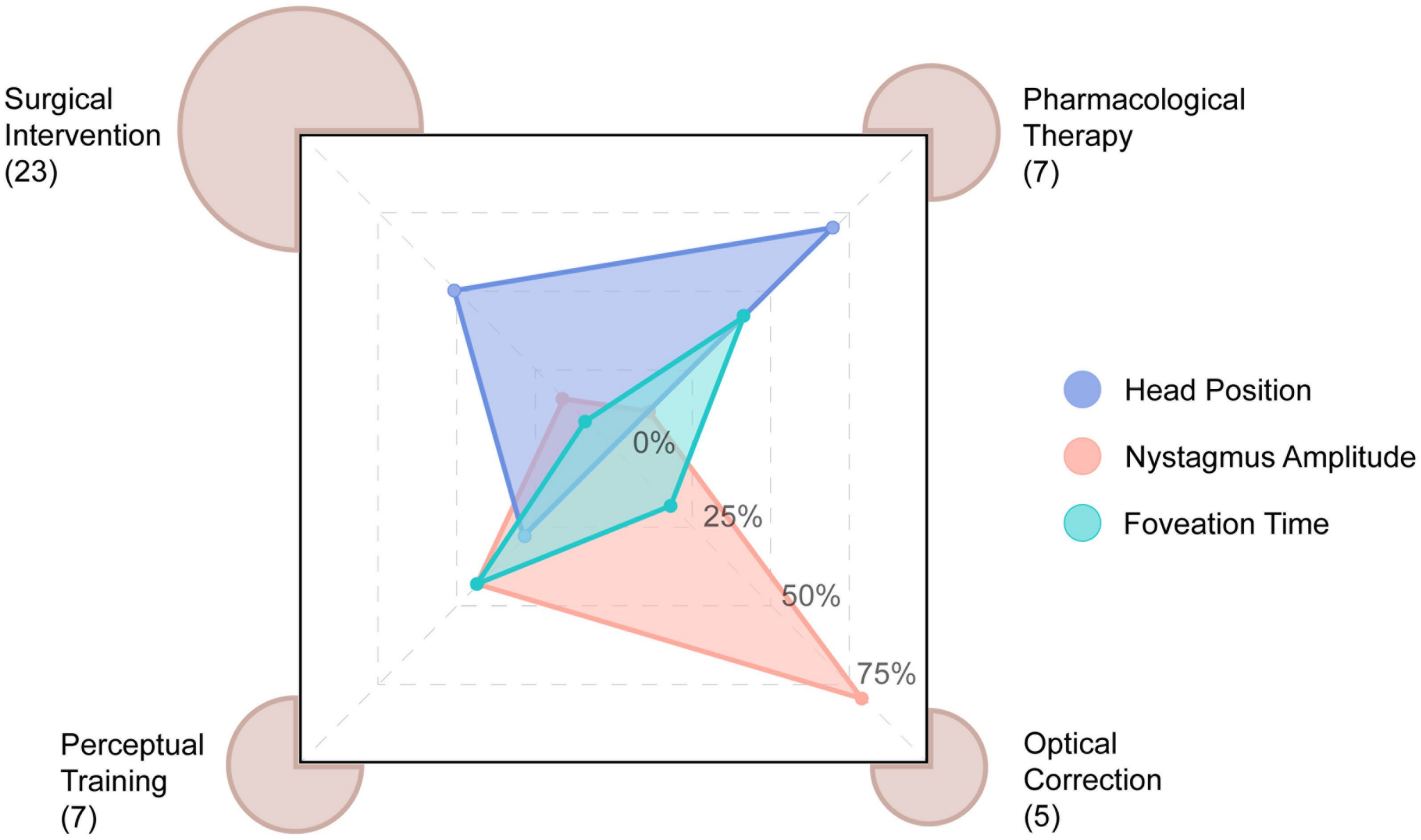
Radar plot illustrating the nystagmus parameters reported across the four treatment classes. Note the substantial heterogeneity in the types of nystagmus parameters reported.

Supplementary Tables 1–4 present information across the four treatment modalities in IN, including the study design, outcomes in VA, nystagmography, QoL, and complications. The 42 journal articles with intervention comprise of randomized control trials (*n* = 7), non-randomized intervention studies (*n* = 14), case–control study (*n* = 1), cross-sectional studies (*n* = 3) and retrospective case report/series (*n* = 17), with no cohort study (*n* = 0). Numbers of different types of studies under each modality can be found in Table 2. Studies often only report specific outcome measures (Figure 2). Significant methodological heterogeneity across the three types of outcomes hinders the comparison of treatment effects between studies (Supplementary Tables 1–4). An example in nystagmus parameters is shown in Figure 3.

**Table 2:** Numbers (n) of different type of studies* under each modality:

Type of intervention	Randomized control trial	Non-randomized intervention study	Case–control study	Cross-sectional study	Retrospective case report/series
Surgical Interventions	1	8	1	0	13
Pharmacological Therapy	1	1	0	3	2
Optical Correction	1	3	0	0	1
Behavioral Training	4	2	0	0	1

^*^No cohort study (*n* = 0).

### 3.1 Surgical intervention

Surgical intervention has been the mainstay of treatment options for patients with IN. Generally, surgeons have to modify the procedure according to the various conditions of patients with associated symptoms. 10 out of 23 reviewed journal articles (43.5%) about surgical treatments (Supplementary Table 1) mentioned potentially associated symptoms in patients with IN, including oscillopsia (Dell’Osso et al., 2018), strabismus (Dell’Osso et al., 2018; Ganesh et al., 2019; Hertle et al., 2021), astigmatism (Fresina et al., 2015; Hertle et al., 2015), myopia (Bagheri et al., 2016), convergence damping (Wang et al., 2015), head tilt (Kekunnaya and Jain, 2022), and AHP (Ganesh et al., 2019; Hertle et al., 2021; Wagdy and Ismael, 2017; Baldev et al., 2022).

Although the procedures are highly individualized, they can be approximately classified into several types based on their strategies:

1. For patients with both IN and AHP, the intervention is designed to move the compensatory head position (CHP) to the primary position. Common approaches include the Kestenbaum procedure and the Anderson procedure. While the Kestenbaum procedure involves bilateral recession of the yoke muscles opposite to the AHP along with bilateral resection or plication of their antagonists, the Anderson procedure only adopts bilateral recession of the muscles (Gräf et al., 2020). Associated variations include the high-dose Anderson procedure (Gräf et al., 2019) and the augmented Anderson procedure (Muralidhar and Ramamurthy, 2021). In general, those interventions can correct or improve AHP (Ganesh et al., 2019; Wagdy and Ismael, 2017; Gräf et al., 2020; Gräf and Lorenz, 2016; Kumar and Lambert, 2018; Zheng et al., 2020), apart from a limited number of cases where no significant change was spotted in AHP (Gräf et al., 2019; Kumar and Lambert, 2018). The effect on VA varied among studies (Supplementary Table 1). However, complications including the slight reverse of the effect (Chen et al., 2019), exophoria (Gräf et al., 2019), strabismus (Zheng et al., 2020), and residual head turn (HT) could happen.
2. For patients with IN whose nystagmus dampens with convergence, one study examined the use of biomedical rectus recession and bilateral rectus tenotomy (Wang et al., 2015). The principle is to reduce nystagmus by generating convergence for binocular vision. Although significant improvements were found in nystagmus frequency and BCVA compared to pre-surgical condition without prisms, the effects are not statistically different from pre-surgical condition WITH prisms.
3. For patients with IN with astigmatism or myopia, photorefractive keratectomy is an evident option for symptomatic treatment. It could also improve VA and reduce nystagmus symptoms (Fresina et al., 2015; Bagheri et al., 2016).
4. The recession, resection, and vertical transposition of the four rectus muscles are also used in IN surgery. The mechanism might be that the inflammation and the scarring affect the nerve fibers from the proprioceptive receptors of the extraocular muscles and change the feedback loop, or that the retreat of the muscles relaxes the muscle itself and shortens the lever arm of contraction (Pan, 2021). Dell’Osso et al. (2016) tested augmented tenotomy and reattachment (AT-R) surgery over the traditional tenotomy and reattachment (T-R) method but found no beneficial improvement with additional sutures. Lingua performed a four-muscle myectomy with pulley fixation, suturing the fascial tissue in front of the pulley structure of the retreating medial rectus muscle to the insertion end of the medial rectus muscle, which effectively alleviated the adduction and secondary exotropia while reducing the nystagmus amplitude and improving the binocular best-corrected visual acuity (BBCVA) and QoL (Lingua, 2020; Lingua and Gore, 2020). Singh et al. (2016) compared two types of surgical methods in a prospective study, showing a trend of improvement in electronystagmography (ENG), contrast sensitivity, and stereopsis.

The main purpose of the surgical options described above is to benefit the patients by damping the characteristics of nystagmus or by correcting torticollis and the goals were met. Besides, VA was tested in 19 (82.6%) out of 23 reviewed articles and 15 (78.9%) out of 19 showed constructive results: 8 articles reported significant improvement in BCVA ranging from 0.037 to 0.21 logMAR; other improvements were shown in contrast sensitivity, corrected distance visual acuity (CDVA) and uncorrected distance visual acuity (UDVA) (Supplementary Table 1). However, real-life performance such as QoL and subjective evaluation of the surgical process was rarely measured in those cases (3 out of 22, 13.6%). Several complications were also reported: no improvement in distance VA (Fresina et al., 2015), annular haze (Fresina et al., 2015), conjunctival cyst (Dubner et al., 2016), remaining oscillopsia (Dell’Osso et al., 2018), exophoria (Gräf et al., 2019; Lingua, 2020), over-and under-corrected AHP or HT (Kekunnaya and Jain, 2022; Zheng et al., 2020), and strabismus (Zheng et al., 2020; Lingua and Gore, 2020).

### 3.2 Pharmacological therapy

Off-label medication was tested in both clinical cases and randomized trials, which include anti-epileptic drugs such as memantine and gabapentin (McLean et al., 2007), and carbonic anhydrase inhibitors such as acetazolamide (Thurtell et al., 2010) and brinzolamide (Hertle et al., 2015; Dell'Osso et al., 2011; Nieves-Moreno et al., 2017; Aygit et al., 2018) (Supplementary Table 2). Carbonic anhydrase inhibitors like brinzolamide may help treat nystagmus by reducing aqueous humor production in the ciliary epithelium, thereby lowering intraocular pressure and modulating neuronal feedback at entheseal sites, which could contribute to stabilization of the ocular motor system (Aygit et al., 2018; Cvetkovic and Perry, 2003; Siesky et al., 2009). The largest (*n* = 48), double-blinded randomized study on anti-epileptic memantine and gabapentin was traced back to 2007 (McLean et al., 2007). After over 55 days of drug intake, several improvements were seen including foveation time, nystagmus intensity, BCVA, and subjective evaluation, along with a long list of side effects including dizziness, tiredness, sleeplessness, forgetfulness, light-headed feeling, depression, nausea, headaches, shakiness, weakness, and drowsiness (McLean et al., 2007). For IIN, gabapentin had no effect while memantine only improved VA by 0.15 logMAR (Richards and Wong, 2015). According to Ospina, memantine has been prescribed to a few of his adolescent patients with IN, and subjective vision improvements were reported (Ospina, 2018). Four studies including one randomized cross-over study over brinzolamide (Azopt) were conducted between 2011 and 2018. The medicine was administered in the form of eye drops and no adverse side effects were reported (Hertle et al., 2015; Dell'Osso et al., 2011; Nieves-Moreno et al., 2017; Aygit et al., 2018). There were improvements in VA but the effect of damping the nystagmus sparsely occurred. A recent study (*n* = 29) from Yadav et al. (2024) further supported the improvement in VA and amelioration of nystagmus acuity by brinzolamide encouraging further investigation. In two other relatively large-scale studies (*n* > 10), several patients (18.1 and 81%) showed or reported no improvement (Nieves-Moreno et al., 2017; Aygit et al., 2018). In the review articles, baclofen, 4-aminopyridine, 3,4-diaminopyridin, clonazepam, diazepam, and botulinum toxin were options to treat nystagmus, but not necessarily for IN (Tegetmeyer, 2014).

### 3.3 Optical correction

Appropriate refractive correction is an essential start to improve VA and amblyopia in IN. More than half of patients with nystagmus (57%) endure significant refractive error and optical correction can sometimes be sufficient to correct them and improve AHP and vision (Papageorgiou et al., 2014; Ospina, 2018). Spectacles, contact lenses, and prisms are common optical approaches. In general, contact lenses improved VA and reduced nystagmus. However, the results varied and quantifications of VA were different (Biousse et al., 2004; Rutner and Ciuffreda, 2005) (Supplementary Table 3). In the latest and largest randomized trial, no significant effect in BCVA was found (Theodorou et al., 2018). The changes in nystagmography were also diverse (Supplementary Table 3).

Bagheri et al. (2017) introduced rigid gas-permeable contact lenses (RGPCL) and they showed promising results in reducing the frequency, intensity, and amplitude of the nystagmus and improving BCVA and contrast sensitivity after 3 months of wearing. According to previous reviews, contact lenses perform better than spectacles (Papageorgiou et al., 2014; Pan, 2021). Some proposed it might be because contact lenses lessen high-order optical aberrations and prismatic effects, continuously align themselves on the visual axis despite eye movement in nystagmus, and stimulate the afferent feedback of the ophthalmic branch of the trigeminal nerve (Ospina, 2018; Pan, 2021). Nevertheless, those arguments remain controversial. Specifically, in an earlier review, McLean claimed no better results in either VA or nystagmography for contact lenses compared to wearing glasses (Papageorgiou et al., 2014). In a recent review, Self et al. (2020) further pointed out the weighing between positive outcomes and contact lenses complication such as infection.

Wang et al. indicated the effect of prisms against surgical intervention in a retrospective clinical study and it turned out that a. the induction of convergence prior to the surgery significantly improved BCVA (from 0.21 ± 0.15 logMAR to 0.28 ± 0.18 logMAR); b. the surgical benefit does not differ from the benefit of prism (Wang et al., 2015). This reported effect in BCVA contradicts with previous literatures (Hertle, 2022; Dickinson, 1986). From a statistician perspective, the improvement in BCVA is not black and white, and further study of randomized, double-blinded trials that systematically analyze this effect across levels of convergence induction are desired. Practically speaking, since prisms are typically used in patients whose convergence would dampen nystagmus, they are naturally not applicable in those lacking binocularity (Ospina, 2018). Furthermore, when prisms are used to correct abnormal gaze, a significant AHP would limit the visual field and thus decrease vision (Ospina, 2018).

### 3.4 Perceptual training

Behavioral training, specifically perceptual training, refers to a process or program designed to enhance and improve individuals’ perceptual abilities and real-life performance (Lu et al., 2011). The interest in perceptual training in the treatment of IN increased relatively later in the timeline (Supplementary Table 4). Its aims of fine-tuning and refining the brain’s ability to receive, process, and interpret sensory information accurately and efficiently have demonstrated potential in treating nystagmus. Huurneman et al. performed a relatively large-scale (*n* = 36), controlled trial investigating the effect of perceptual training on children with IN in 2016. After either crowded or uncrowded computer-based discrimination tasks, nystagmus characteristics, VA, and reading performance were examined. The results showed that there was no effect on damping the nystagmus, but after training improvements in VA were seen which were associated with improvements in reading acuity and critical print size (Huurneman et al., 2016a; Huurneman et al., 2016b; Huurneman et al., 2016c).

Another clinical trial carried out by Mohamad Fadzil et al. (2019) showed distinct results, where AHP and reading performance improved but VA and contrast sensitivity did not change significantly. One explanation could lie in the difference in the training scheme since a comprehensive perceptual task containing feedback would be considered more compelling in adolescence, thus increasing focus from the participants and potential effect on the training. In 2021, Goossens and Huurneman conducted a prolonged home-based perceptual training study in children and adolescents (*n* = 37), where VA, stereopsis, single letter acuity, crowded acuity, and crowding extent improved significantly (Huurneman and Goossens, 2021). There were no changes in reading acuity and critical print size, but reading speed improved 2 × more than expected by natural development (Huurneman and Goossens, 2021). Enhancements were also observed in school activities and mobility activities (Huurneman and Goossens, 2021).

Another type of perceptual training is biofeedback training, which is a process that involves using electronic or physiological sensors to provide individuals with real-time information about their performance, allowing them to adjust their regulation techniques (Vingolo et al., 2018; Deng et al., 2023). Daibert-Nido et al. (2021) and Caputo et al. (2021). investigated the effect of biofeedback training in patients with IN, respectively, but the results in VA were different. The former detected significant improvements in VA, stereopsis, contrast sensitivity, and reading speed while the latter showed no improvement in VA but only in fixation ability indicated by bivariate contour ellipse area (BCEA). A longitudinal study with no intervention in patients with IIN revealed an improvement in VA over time: 0.16 logMAR per 10 years, with follow-ups up to 18 years (Balzer et al., 2018). Furthermore, a prognostic influence on the impact of perceptual learning in children with IN was identified by a study, indicating age and baseline performance as the relevant factors, which might contribute to the diverse results of perceptual training in IN (Huurneman et al., 2017).

## 4 Discussion

The present review comprehensively examines the current state of research on the symptomatic treatment of IN. By critically evaluating the available evidence, this discussion aims to shed light on the effectiveness, limitations, and future directions of various treatment approaches. In this section, we will delve into the main findings, address unresolved questions, and explore the implications of these findings for clinical practice and future research.

Broadly speaking, the primary objective of surgical and pharmacological interventions (Supplementary Tables 1, 2) is to reduce nystagmus, whereas the main goal of optical and behavioral approaches is to enhance visual function (Supplementary Tables 3, 4). In the case of the former interventions, the observed improvements in VA were relatively modest. However, there are indications that visual functions, such as recognition time, contrast sensitivity and motion processing improve by improving the nystagmus waveform, even if VA does not increase measurably (Käsmann-Kellner, 2017). Based on the gathered information, surgical intervention remains the predominant treatment modality for addressing IN. It was advised that it should be avoided in the cases of children under 5 years old because the pre-surgery measurement and assessment are challenging due to a lack of cooperation (Käsmann-Kellner, 2017). Meanwhile, surgery on patients under 2 years old demonstrated promising outcome in ocular motor and visual system (Hertle et al., 2009). Even though there are multiple surgical methods, there is no targeted pathology. The same situation also stands in medication. Currently, most of the pharmaceutical studies are off-label and there was no study on the long-term effect of medication for IN, which impeded its clinical use (Ospina, 2018). Additionally, the pharmacological treatment for nystagmus is typically reserved for adults who present with distressing visual symptoms, such as oscillopsia. Given the rarity of these symptoms in early-onset nystagmus, pharmacological intervention is often deemed unnecessary (Richards and Wong, 2015). Among the medications examined, brinzolamide exhibited promising outcomes in enhancing VA, with the added advantage of its administration via eyedrops, which contributes to its safety profile (Hertle et al., 2015; Dell'Osso et al., 2011; Nieves-Moreno et al., 2017; Aygit et al., 2018; Yadav et al., 2024). As previously mentioned, a longitudinal study without any intervention demonstrated a slight improvement in VA throughout a lifetime tested before 18 (Balzer et al., 2018). This observation naturally leads us to think that the self-correcting influence of daily experiences may have a positive impact on individuals with IN and perceptual training can be seen as an accelerated form of this natural process. The reduction in population receptive field (pRF) size in V2 and the alleviation in visual orientation crowding provided additional evidence supporting the efficacy of perceptual training and its impact on neuronal plasticity (He et al., 2019). However, as the measurement results (e.g., VA) vary among individuals and the follow-up periods in clinical cases are often limited, we also suggest researchers take caution and do not rely solely on the significance (e.g., *p*-value) of a univariate approach (Morey et al., 2016).

Although we provide a systematic review of recent symptomatic IN treatments, the articles reviewed had several overarching limitations. Firstly, there was a lack of standardization in outcome measurements (Figures 2, 3, Supplementary Tables 1–4). Due to the heterogeneity of the outcome measurements, conducting a quantitative comparison of treatment types or cross-benefit analysis was impossible. Different units or measurements for certain parameters, such as VA represented by either logMAR units, Snellen fraction, Snellen line gain, or decimal acuity, complicated the comparison between studies and treatment options (Wesemann et al., 2020) (Supplementary Tables 1–4). The eXpanded Nystagmus Acuity Function (NAFX) index has been proposed as a measure of VA (Khanna and Dell'Osso, 2006). However, there is evidence of limitations in using NAFX and other eye movement measurements for patients with apparent visual issues and its inefficacy in patients with albinism (Hertle and Dell’Osso, 2023). Moreover, those measurements are not often direct indicators of visual function (Hertle and Dell’Osso, 2023). The main goal of symptomatic treatments of nystagmus is to improve the QoL of the patients, raising the question of whether more emphasis should be placed on patients’ well-being and real-life performance or their nystagmus signs. Even though the reduction the nystagmus intensity could improve QoL via improving visual functions, the QoL evaluation was absent in most of the studies other than perceptual learning (Figure 2). We recommend that clinicians prioritize the primary objective of improving the overall well-being and daily functioning of patients, assess and select treatment options accordingly, and report QoL evaluation and related functional factors such as binocular VA, gaze dependent VA, contrast sensitivity, visual task performance including recognition time, etc. For future trials and clinical studies, it is highly suggested that we include, and report detailed settings in a unified way. One example would be, for VA, specifying near or distant and crowded or non-crowded VA would different aspects of participants’ sensory states. This would also help further quantitative comparison across studies since if the test methods are sparse, the analysis would be underpowered. Moreover, only after a systematic report of standardized nystagmus measurements, questions such as which nystagmus trait affects VA the most could be studied.

The second limitation of the reviewed articles is that most of the studies were clinical trials with small sample sizes and short follow-up times. More long-term studies are needed in the field since a lasting effect or an adverse effect would largely alter the benefit of the intervention (Fossataro et al., 2024). In that way, if the effect is similar, non-surgical intervention would be preferred which contradicts the status quo.

The third limitation of the current research is the lack of information of personalization and categorization of the patients. IN is complicated and it relates to multiple other illnesses which makes handling of screening and diagnosing data across studies a challenge. If a systematic report of personalization and categorization procedure were available in every study, a better insight into which intervention would fit which type of patients would be feasible when researchers and clinicians line up the inclusion criteria and treatment methods (Self et al., 2020; Käsmann-Kellner, 2017).

It is nevertheless important to note that many of the articles reviewed were retrospective studies, and there may be some overlapping in the statistics presented. We classified them into different categories to give general insight into treatment options, but they can be essentially divergent. Furthermore, this review exclusively includes articles concerning studies in humans, while nystagmus models in zebrafish and mice could offer valuable insights into the fundamental nature of the disease (Bögli et al., 2017; Winkelman et al., 2019). On the other hand, integrating mechanical (Beh et al., 2014) or magnetic (Nachev et al., 2017) force has shown potential in dampening acquired nystagmus, which can also be inspiring for future applications for IN treatment. Lastly, it is often unclear whether the articles examined have adopted the definition of IN as outlined by the Classification of Eye Movement Abnormalities and Strabismus (CEMAS) Working Group in 2001, which distinguishes it from other types of early-onset nystagmus such as fusion maldevelopment nystagmus syndrome (FMNS), spasmus nutans syndrome, etc. (CEMAS, 2001). Readers should be mindful of this consideration.

## 5 Conclusion

Methodological heterogeneity in outcome measures is ubiquitous in the current research field of symptomatic treatments of infantile nystagmus. We conclude that the field needs to adopt standardized outcome measures to enable quantitative comparison of intervention approaches and meta-analyses. To support patient well-being, we further suggest that functional vision measurements and quality of life should be included in future research.

## Data Availability

Publicly available datasets were analyzed in this study. This data can be found at: PubMed Queries mentioned in the article.
