# Supplementary material for: Symptomatic treatment of infantile nystagmus: a systematic review
Source: Front Neurosci. 2025 Jun 19;19:1612504. doi: 10.3389/fnins.2025.1612504 (PMC12222088; doi:10.3389/fnins.2025.1612504)
Supplement: Supplementary file 1 [file Table_1.docx]

Supplementary Material

# Supplementary Table 1. Summary of recent evidence on surgical interventions for IN.

| **Reference** | **Study type** | **Number of participants** | **Age range (y)** | **Diagnoses** | **Type of intervention(s)** | **Treatment procedure** | **Improvement in nystagmography and gaze / posture** | **Changes in VA / NAFX** | **Changes in QoL & everyday life functioning** | **Follow-up after** | **Complications & notes** |
| --- | --- | --- | --- | --- | --- | --- | --- | --- | --- | --- | --- |
| Wang, Y., Wu, Q., Bai, D., Cao, W., Cui, Y., Fan, Y., Hu, S., & Yu, G. (2015). | retrospective case series | 8 | 9.5 (6-12) | IN (100%), convergence damping (100%) | surgical + optical | bimedial rectus recession and bilateral rectus tenotomy  prism | frequency (p<0.05);  amplitude, no significance | Method: standard  Distance: not specified  Primary \| null: primary  Δ VA = 0.07 logMAR (prism)  Δ VA = 0.11 logMAR (surgery) | N/A | 9 (6-24) mos | BCVA from surgery is not significantly different to prism. |
| Fresina, M., Giannaccare, G., Gizzi, C., Versura, P., & Campos, E. C. (2015). | retrospective case series | 11 | 30.8 (22-42) | IN (100%), astigmatism (100%), oculocutaneous albinism (18%) | surgical | photorefractive keratectomy | N/A | Method: not reported  Distance: distance (corrected (pre) and uncorrected DVA (post))  Primary \| null: null  Δ CDVA = 0.08 logMAR | Postoperative patient satisfaction questionnaire (score range 0 (no satisfaction) – 6 (max. satisfaction)).  Mean satisfaction 5.3/6 | 1 y | 6 (55%) patients with previous Anderson operation; 2 eyes with worse UDVA than preoperative CDVA; no vision-threatening complication; 1 patient with annular haze |
| Yang, H., Yu, T., Yao, J., Tai, Z., Wang, M., & Yin, Z. (2015). | non-randomized intervention study | 16 | 16 (6-20) | IIN (100%):  - horizontal impulsive nystagmus (87.5%), horizontal pendulum nystagmus (12.5%), AHP (81%), strabismus (19%), convergence damping (6%) | surgical | Parks (patients with horizontal AHP);  Anderson-Kestenbaum (patients with vertical AHP);  divergence (patient with convergence damping);  T-R (patients with no AHP);  fixation surgery (patients with strabismus) | horizontal intensity improved in 9 patients (67.63%, p<0.01);  vertical intensity improved in 5 patients (69.94%, p<0.05) | N/A | N/A | 9.8 mos (3w - 33mos) | evaluated using digital eye tracker |
| Bagheri, A., Abbasi, H., Tavakoli, M., Baradaran-Rafii, A., Shaibanizadeh, A., Kheiri, B., & Yazdani, S. (2016). | non-randomized intervention study | 12 | 23±2 | IN (100%) and myopia (100%) | surgical | photorefractive keratectomy | frequency -0.92 Hz (P <0.01);  amplitude -11.75 mV (P <0.01);  intensity -38.70 degree/s (P <0.01) | Method: standard Snellen chart, 6m; mono- and binocular  Distance: 6m  Primary \| null: null  Δ VA (monocular) =0.09logMAR  Δ VA (binocular) = 0.16 logMAR | N/A | 3 mos | N/A |
| Dubner, M., Nelson, L. B., Gunton, K. B., Lavrich, J., Schnall, B., & Wasserman, B. N. (2016). | non-randomized intervention study | 15 | 15 (3-77) | 9 (60%) IN, 0 strabismus, 0 AHP | surgical | four-muscle tenotomy | intensity derease (P = .043) | Method: not specified  Distance: distance, bilateral distance, and near  Primary \| null:not speficied  Δ VA (binocular) = 0.12 logMAR | perceived vision im- provement (87%); perceived decrease in nystagmus intensity (73%); perceived depth perception improvement (53%); other benefits (47%) | 8.6 ± 4.9 mos | 1 (7%) conjunctival cyst |
| Gräf, M., & Lorenz, B. (2016). | retrospective case series | 11 | 7 (4-30) | IN (100%), IIN (45.5%), sensory defect nystagmus (45.5%), optic nerve atrophy (9%) | surgical | the Anderson procedure (equal recessions on the horizontal yoke muscles opposite to the AHP of 12 mm (10-17)) | AHP reduced to 7° (0°-20°) (3-6 mos); AHP ≤ 10° (73%); AHP ≤ 15° (82%);  esophoria in 1 patient reduced from 7° to 2° | Method: mono- and binocular VA, method not reported  Distance: not reported  Primary \| null: null  VA unchanged | N/A | 3-25 mos | 1 case using bovine pericardium grafts; 2 patients later received augmenting surgery |
| Singh, A., Ashar, J., Sharma, P., Saxena, R., & Menon, V. (2016). | randomized controlled trial | 10 | ≥ 5 | IN with null point (100%) | surgical | group 1: Retroequatorial recession;  group 2: Hertle-Dell’Osso procedure | no significant results (p>0.05) | Method: Snellen and logMAR charts.  Distance: distance.  Primary \| null: null  VA unchanged | N/A | N/A | N/A |
| Dell’Osso, L. F., Orge, F. H., & Jacobs, J. B. (2016). | non-randomized intervention study | 4 | 8-55 | IN (100%) | surgical | augmented tenotomy and reattachment surgery (AT-R) | LFD +127%, +122+%, +12+%, N/A | Method: Snellen decimal, NAFX  Distance: not specified.  Primary \| null: primary  Outcome improvements in NAFX vary but are close to ones from single-suture data. | Qualitative reports (mostly positive) | N/A | not a better alternative to traditional T-R surgery |
| Wagdy, F. M., & Ismael, M. E. (2017). | non-randomized intervention study | 15 | 4-10 | IN with null point (100%), AHP (93,3%) | surgical | augmented modified Kestenbaum procedure | number of AHP from 14 (93.3%) to 3 (20%) (p=0.044); ocular alignment (p≤0.001) | Method: E-game  Distance: not specified.  Primary \| null: primary and null (only at T0).  Δ VA = 0.30 (unit unclear, primary) | N/A | 29 mos | successful surguries in 87.5% patients |
| Dell’Osso, L. F., Orge, F. H., Jacobs, J. B., & Wang, Z. I. (2018) | non-randomized intervention study | 3 | 0.1, 2, 14 | IN (67%-100%); strabismus (67%); | surgical | multiple, including Kestenbaum null-point correction surgery | LFD +23% | No report of VA improvements.  Peak NAFX +23% | N/A | 7-43 y | N/A |
| Dell’Osso, L. F., & Huang, S. S. (2018). | retrospective case report | 1 | 74 | IN (100%) | surgical | retina post-removal of an epiretinal membrane | focal oscillopsia less noticeable | Method: not specified  Distance: not specified  Primary \| null: not specified  Δ VA improved from 20/40 to 20/25 | N/A | 2 y | oscillopsia remained |
| Kumar, P., & Lambert, S. R. (2018). | retrospective case series | 7 | 0.33-5 | IN (100%), albinism (57%), IIN(29%), cone-rod dystrophy (14%) | surgical | extraocular muscle surgery, separate Kestenbaum procedure (14%) | 3 with corrected AHP; 3 with improved AHP (mean 25° vertically); 1 with no siginifance; | Method: Snellen charts  Distance: not specified  Primary \| null: null  The authors were unable to extrapolate direct visual acuity  benefits from the surgical intervention, because of underlying ophthalmic diagnoses and young age of children. | N/A | 8 (1-9.5) y, median | N/A |
| Ganesh, S. C., Rao, S. G., & Narendran, K. (2019). | retrospective case series | 37 | 12.39 ± 8.64 | IIN and eccentric null zone presenting with AHP (100%), associated strabismus (5.41%) | surgical | modification of Anderson’s procedure, correction for strabismus for strabismus patients | AHP from 22.5° ± 6.12° to 7.58° ± 3.62° (p<0.001) | Method: Snellen or Sheridan Gardner chart  Distance: near+distance  Primary \| null: both  Only BCEA for primary position at distance was compared. No report of VA changes in null position or near vision.  Δ DVA = 0.22 logMAR | N/A | 1 mos | one patient with a second surgery (resection of yoke muscles). |
| Chen, J. J., Tian, L. L., Zhang, L. H., Wang, J. L., & Kang, X. L. (2019). | retrospective case series | 17 | 5-11 | IN (100%) | surgical | null zone shift surgery: Parks 5⁃6⁃7⁃8, Anderson, Kestenbum 5⁃5⁃6⁃4,null zone shift combined with strabismus correction and vertical null zone transposition | foveation time no significance;  reduction in the null zone position P=0.000 | Method: Snellen  Distance: not specified  Primary \| null:primary and null  Δ BCVA ~ 0.06 logMAR  NAFX no significance | N/A | 14.8 ± 6.0 mos | 6 (35%) showed slight reverse in reduction in the null zone position |
| Gräf, M., Hausmann, A., & Lorenz, B. (2019). | non-randomized intervention study | 29 | 7 (4-44) | IN (100%), IIN (59%), foveal hypoplasia (17%), albinism (10%) | surgical | high-dose Anderson procedure | AHP corrected;  AHP components in vertical and frontal planes  with no improvement | Method: Landolt C or LEA symbols  Distance: 0.3 and 5m  Primary \| null: null  Δ DVA = 0.04 logMAR (n.s.) | N/A | ≥8 mos (24 patients) | exophoria in 2 patients; 3 drop outs; 1 early 2nd surgery |
| Gräf, M., Hausmann, A., Kowanz, D., & Lorenz, B. (2020). | retrospective case series | 52 | 4-44 | IN (100%) | surgical | 33 with Anderson procedure; 19 with Kestenbaum procedure | AHP reduction 75% (Anderson), 70% (Kestenbaum);  AHP reduction long-term 67% (Anderson), 64% (Kestenbaum) | Method: not reported  Distance: not reported  Primary \| null: null  Δ DVA < 0.1 logMAR | N/A | 14 mos (8-61, Anderson); 36 mos (15-153, Kestenbaum) | N/A |
| Lingua, R. W. (2020). | retrospective case series | 2 | 4, 28 | IN (100%) | surgical | subject 1: myectomy of the four horizontal rectus muscles; reoperation for exotropia;  subject 2: myectomy with pulley fixation | Amplitude -80% and -100% | Method: for the young girl Allen figures before and Snellen letters 4y post-op.  Distance: near+distance  Primary \| null:null  BBCVA  subject 1: 20/125 to 20/100 (distance); 20/150 to 20/80 (13 inches with 4y lapse), after second surgery no change in VA at distance and 13 inches.  subject 2: No changes in distance VA; at near (13 inches) 0.2 logMAR improvement 20/40 to 20/16 | N/A | 4 y and 11 mos | 1 (50%) residual exotropia after the first surgery; |
| Lingua, R. W., & Gore, C. (2020) | case-control study | 10 | 31 (18-42) | AHP (20%), DysChr (10%), FH (50%), FMNS (10%), IN (80%), partial optic atrophy (10%), OcCutAlb (20%)  No-HF = Group without foveal hypoplasia (n=5)  FH = group with foveal hypoplasia (n=5) | surgical | four-muscle myectomy with pulley fixation | amplitude -69% (FH), -32% (no FH);  frequency -50% (FH), -23% (no FH);  aSPV distance -47% (FH), -65% (no FH);  foveation time +366% (FH), +67% (no FH) | Method: Snellen chart  Distance: 6m, 0.8m and 0.35m  Primary \| null: null  No-FH group  Δ VA 6m = 0.15 logMAR (median)  Δ VA 0.8 and 0.35m = 0.1 logMAR (median)  FH group  Δ VA = 0.05 logMAR  Δ VA 0.8m = 0.05 (median) logMAR (median)  Δ VA 0.35m = 0.1 logMAR (mean) | Adult Nystagmus Quality of Life Questionnaire (NYS-29) | 6 mos | 5 (50%) induced strabismus,  5 (50%) with loss of adduction |
| Zheng, Y., Law, J. J., Holt, D. G., Morrison, D. G., & Donahue, S. P. (2020). | retrospective case series | 150 | Mean age = 8.1y | nystagmus (100%), IIN (59%), sensory abnormalities (38%), acquired nystagmus (3%) | surgical | 31 with surgery for AHP in the pitch (chin up/down) position; 119 with surgery for a horizontal AHP | corrected (within 10°) AHP from 95% (1-3 w, n=132) to 82% (2 y, n=57) to 93% (5 y, n=42) to 93% (10 y, n=14) | Method: not specified  Distance: not specified  Primary \| null: null  Δ DVA n.s. | N/A | 1 w - 10 y | over- vs undercorrected: 5 vs 0% (1-3 w), 11 vs 7% (2 y), 0 vs 7% (10 y); strabismus 6.7% |
| Hertle, R. W., Curtis, M., Boydstun, I., Juric, A., Evliyaoglu, F., & Ricker, I. (2021). | non-randomized intervention study | 75 | 36 (18-72) | IN (100%), albinism (35%), amblyopia (23%), optic nerve or retinal disorders (48%), refractive error (80%), AHP (44%), aperiodicity (27%), and strabismus (69%) | surgical | a surgical algorithm of nine separate procedures | siginifance in AHP, strabismic deviation | Method: E-ATS, NAFX  Distance: unknown  Primary \| null: null  BCVA 0.14 logMAR (p < 0.01)  NAFX 18.5%-24% (p < 0.01) | N/A | 14 (13-78) mos | 12% reoperation rate for recurrent strabismus or  recurrent AHP |
| Muralidhar, R., & Ramamurthy, D. (2021). | retrospective case series | 8 | 14 (6-24) | IN (100%) | surgical | plication augmentation of the augmented Anderson procedure | no induced strabismus;  mean correction in head turn 25° ± 6.5° with median prismatic correction 45PD (3 fully corrected; all <10°) | Method: not specified  Distance: not specified  Primary \| null: null  No statistical comparison or descriptives of visual acuities before and after. | N/A | ~ 1 mos | two patients with surgery for a residual head turn. |
| Baldev, V., Tibrewal, S., Rath, S., & Ganesh, S. (2022). | retrospective case report | 1 | 26 | IN (100%), AHP (100%) | surgical | four horizontal rectus muscle recession and resection with full tendon vertical transposition | AHP improved significantly; chin depression reduced | N/A | N/A | 1.5 y | vertical diplopia; depression limitation; elevation limitation |
| Kekunnaya, R., & Jain, M. (2022). | retrospective case series | 3 | 4-7 | IN (100%), head tilt (100%) | surgical | vertical transposition of four horizontal rectus muscles | head tilt reduced | N/A | N/A | N/A | 1 with residual head tilt |

AHP, anomalous head posture; aSPV, average slow phase velocity (degree/s); AT-R, augmented tenotomy and reattachment surgery; BBCVA, binocular best-corrected visual acuity; BCVA, best corrected visual acuity; CDVA, corrected distance visual acuity; DysChr, dyschromatopsia (loss of red-green); FH, foveal hypoplasia; FMNS, fusion maldevelopment nystagmus syndrome; IN, infantile nystagmus; IIN, idiopathic infantile nystagmus; LFD, longest foveation domain (the second ‘+’ indicating endpoints >±30°); MBCVA, monocular best-corrected visual acuity; NAFX, eXpanded Nystagmus Acuity Function; OCCutAlb, oculocutaneous albinism; QoL, quality of life; T-R, tenotomy and reattachment surgery; UDVA, uncorrected distance visual acuity; VA, visual acuity; w/mos/y, week(s)/month(s)/year(s)

# Supplementary Table 2. Summary of recent evidence on pharmaceutical treatments for IN.

| **Reference** | **Study type** | **Number of participants** | **Age range (y)** | **Diagnoses** | **Type of intervention(s)** | **Treatment procedure** | **Improvement in nystagmography and gaze / posture** | **Changes in VA / NAFX** | **Changes in QoL & everyday life functioning** | **Follow-up after** | **Complications & notes** |
| --- | --- | --- | --- | --- | --- | --- | --- | --- | --- | --- | --- |
| McLean, R., Proudlock, F., Thomas, S., Degg, C., & Gottlob, I. (2007). | randomized controlled trial | 48 | ~39.8 | IN (100%)  IIN (44.7%)  Secondary Nystagmus (55.3%) | pharmaceutical | dynamic dosages of memantine, gabapentin, and placebo in 35 days following by a constant dosage for 21 days (memantine 40mg; gabapentin 2400mg) | foveation using NAFX memantine: (p=0.001) gabapentin: (p=0.02);    Intensity memantine: (p=0.001) null region (p=0.02) all positions; gabapentin: (p=0.01) null region (p=0.14) all positions | Method: EDTRS Sloan letters  Distance: not specified  Primary \| null: null  Memantine:  CIN: Δ VA 0.15 ± (SD) 0.18 logMAR  SN: Δ VA 0.05 ± 0.04  Gabapentin:  CIN: Δ VA 0.09 ±0.05  SN: Δ VA 0.04 ± 0.07  Placebo:  CIN: Δ VA: 0.04 ± 0.03  SN: Δ VA -0.03 ± 0.05 | reported  subjective improvement [in VA] memantine: 10; gabapentin: 9; placebo  1 (p=0.03);  [in nystagmus] memantine: 7; gabapentin: 6; placebo  1 (p=0.17);  VF-14 memantine:37 to 28%, gabapentin: 24 to 17.5%, placebo: 34% to 28% (p=0.50)  SFQ memantine: 71 to 76.5%, gabapentin: 73 to 80.5%, placebo: 67.5% to 74% (p=0.95) | 3 mos; 13 patients continued mematine for 9 mos; 13 patients continued gabapentin for 10 mos after the study | side effects including dizzy, tired, sleepless, forgetful, light-headed, depressed, nauseated, headaches, shaky, weak, and drowsy; VA deteriorated 2 w after the stop of the medication |
| Thurtell, M. J., Dell’Osso, L. F., Leigh, R. J., Matta, M., Jacobs, J. B., & Tomsak, R. L. (2010). | cross-sectional study | 1 | 68 | IN | pharmaceutical | 500mg of acetazolamide twice a day for three days (oral) | LFD from 20° to 34°; 70% broadening of the NAFX vs Gaze Angle curve | No vision chart measurements.  peak NAFX 59.7% | N/A | N/A | transient paraesthesia of the hands, abdominal discomfort,“general, mild discomfort” |
| Dell'Osso, L. F., Hertle, R. W., Leigh, R. J., Jacobs, J. B., King, S., & Yaniglos, S. (2011). * | non-randomized intervention study | 1 | 68 | IN | pharmaceutical | 1 drop of topical brinzolamide (Azopt) 3 times daily in each eye on days 1-3 | LFD from 20° to 30°; 50% broadening of the NAFX vs Gaze Angle curve | No vision chart measurements.  peak NAFX 51.9% | N/A | N/A | data from corrigendum |
| Hertle, R. W., Yang, D., AdkINon, T., & Reed, M. (2015). | cross-sectional study | 5 | 33-70 | IN (100%), strabismus (80%), myopia (20%), amblyopia (60%), staphyloma (20%),  astigmatism (60%), amblyopia (60%) | pharmaceutical | self-administered either topical brinzolamide (Azopt) or placebo every 8 h in both eyes for 3 days, procedure swapped after a washout | improved IN waveform characteristics in the null zone after Azopt; | Method: EDTRS protocol bino- and monocularly  Distance: not specified  Primary \| null: null  Median change : Δ 0.12 logMAR  NAFX improved significantly (p < 0.01);  VA from logMAR 1.1 to logMAR 0.98 (p<0.001) | N/A | N/A | no cases of adverse effects |
| Nieves-Moreno, M., Fernández, L. M., Gordo, B. D., Maillo, E., Diaz, E., & Gomez-de-Liano, R. (2017). | retrospective case series | 11 | 6-44 | IN (100%), ocular albinism (27%), albinism (9%), myopia (9%), strabismus (9%), exophoria (9%) | pharmaceutical | topical brinzolamide (Azopt) | frequency almost no sigificance;  intensity almost no sigificance | Method: Snellen chart  Distance: 5m and 0.35m  Primary \| null: null  BCVA distance, no significant changes (<0.05 logMAR).  BCVA near small improvement (median change 0.09 logMAR). | 9 (81%) with no notice of improvement; 2 (18%) with minimum VA improvement; 1 (9%) with less head oscillation | N/A | 1 patient with myopia went through strabismus surgery befofre |
| Aygit, E. D., Ocak, O. B., İnal, A., Fazıl, K., Akar, S., & Gokyigit, B. (2018). | retrospective case series | 23 | 12.6 ± 5.5 | IIN (78.2%), oculocutaneous albinism (21.8%) | pharmaceutical | eye drop of topical brinzolamide (Azopt) in both eyes for 3 days | reduced nystagmus amplitude in 5 (22.7%) patients; reduced AHP in 4 (18.3%) patients | Method: not specified.  Distance: near and distant (unspecified).  Primary \| null: null  Mean VA change 0.03 logMAR across 23 patients. No specification of magnitude of near and distance VA changes. | N/A | 32 ± 28 mos | no change in 4 (18.1%) patients |
| Yadav, B., Saxena, R., Dhiman, R., Kochhar, K. P., Patil, A., Sharma, P., Sihota, R., & Tandon, R. (2024). | cross-sectional study | 29 | 17.65 ± 7.22 | IIN (100%), AHP (79.3%) | pharmaceutical | topical brinzolamide or placebo for 3 months | mean IOP reduction following brinzolamide: 1 ± 0.64 mmHg in group 1 and 0.83 ± 0.36 mmHg in group 2 (p = 0.01); no significant effect noted for AHP or near stereoacuity | Method: ETDRS chart  Distance: 4m  Primary \| null: primary  NAFX improved significantly (p < 0.001); Δ VA ~ 0.065 logMAR | N/A | 3 d | After the wash-off period, VA in group 2 returned to the level before the treatment |

AHP, anomalous head posture; BCVA, best corrected visual acuity; IN, infantile nystagmus; IIN, idiopathic infantile nystagmus; LFD, longest foveation domain; IOP, intraocular pressure; NAFX, eXpanded Nystagmus Acuity Function; QoL, quality of life; SFQ, social function questionnaire; VA, visual acuity; VF-14, Visual Function 14 questionnaire (0% = can perform all 14 visual tasks); *, data from corrigendum; w/mos/y, week(s)/month(s)/year(s); note: the follow-up duration signifies the time after the intervention has ceased.

# Supplementary Table 3. Summary of recent evidence on optical correction for IN.

| **Reference** | **Study type** | **Number of participants** | **Age range (y)** | **Diagnoses** | **Type of intervention(s)** | **Treatment procedure** | **Improvement in nystagmography and gaze / posture** | **Changes in VA / NAFX** | **Changes in QoL & everyday life functioning** | **Follow-up after** | **Complications & notes** |
| --- | --- | --- | --- | --- | --- | --- | --- | --- | --- | --- | --- |
| Biousse, V., Tusa, R. J., Russell, B., Azran, M. S., Das, V., Schubert, M. S., ... & Newman, N. J. (2004). | non-randomized intervention study | 4 | ≥18 | IN (100%) | optical | contact lenses | mean peak amplitude, peak velocity, LFD :  1 improved, 1 worsened, 2 no change;  frequency no change | Method: Snellen, EDTRS  Distance: not specified  Primary \| null: ‘patients with a null point were asked to avoid their null point during evaluation’.  Mean Δ VA = 0.20 logMAR | mean VFQ-25 from 64.7 to 72.05 | N/A | N/A |
| Rutner, D., & Ciuffreda, K. (2005). | non-randomized intervention study | 1 | 18 | IN, ocular albinism | optical | contact lenses | amplitude from 9° to 1.75°, to 0.72° (1 w); frequency from 1.25 Hz to 1.05 Hz, to 1.80 Hz (1 w) | Method: Snellen chart high contrast and Bailey Lovie low contrast VA  Distance: not specified  Primary \| null: null  Baseline binocular VA and low contrast not measured/reported.  High contrast  Δ VA OD: 0.07 logMAR  Δ VA OS: 0.20 logMAR  Some further improvement at 1-week follow up. | N/A | 1 w | N/A |
| Bagheri, A., Abbasi, H., Tavakoli, M., Sheibanizadeh, A., Kheiri, B., & Yazdani, S. (2017). | non-randomized intervention study | 16 | 18.6 ± 4.9 | IN (100%), hyperopia (43.7%), albinism (37.5%) | optical | RGPCL for more than 6 hours a day for at least 3 months | frequency -0.6 Hz (p<0.001);  amplitude -7.8 mV  (p<0.001);  intensity -49.9  (p<0.001) | Method: Snellen chart  Distance: 6m  Primary \| null: null  Δ VA: 0.04 logMAR | N/A | N/A | amblyopia-prone, strabismus, AHP patients excluded |
| Theodorou, M., Quartilho, A., Xing, W., Bunce, C., Rubin, G., Adams, G., & Dahlmann-Noor, A. (2018). | randomized controlled trial | 38 | 18-64 | IN (100%) | optical | fully corrective contact lenses or plano contact lenses (patients who completed CL wear for at least 2 weeks) | mean amplitude -1.19° (plano, p<0.05);  velocity -2.67°/s (corrective, p<0.05) | Method: ETDRS chart  Distance: 4m and near  Primary \| null: null  Δ VA plano CL group: 0.07 logMAR (95% CI 0.03 to -0.11 logMAR change) n.s.  Δ VA corrective CL group: 0.06 logMAR (95% CI 0.02 to -0.1 logMAR change) n.s. | N/A | 14 ± 3 days | 3 ppl with contact lenses discomfort; 3 cases of contact lenses tearing; no keratitis |
| Fossataro, C., Pafundi, P. C., Mattei, R., Cima, V., De Rossi, F., & Savino, G. (2024). | retrospective case series | 20 | N/A | IN(100%) | optical | occlusion in 15 subjects  prism in 5 subjects | no difference from the therapeutic approach | no difference from the therapeutic approach | N/A | N/A | N/A |

AHP, anomalous head posture; BCVA, best corrected visual acuity; BBCVA, binocular best-corrected visual acuity; CDVA, corrected distance visual acuity; IN, infantile nystagmus; LFD, longest foveation domain; MBCVA, monocular best-corrected visual acuity; NAFX, eXpanded Nystagmus Acuity Function; QoL, quality of life; RGPCL, rigid gas-permeable contact lenses; VA, visual acuity; VFQ-25, quality of life questionnaire; w/y, week/year(s); note: the follow-up duration signifies the time after the intervention has ceased.

# Supplementary Table 4. Summary of recent evidence on behavioral training for IN.

| **Reference** | **Study type** | **Number of participants** | **Age range (y)** | **Diagnoses** | **Type of intervention(s)** | **Treatment procedure** | **Improvement in nystagmography and gaze / posture** | **Changes in VA / NAFX** | **Changes in QoL & everyday life functioning** | **Follow-up after** | **Complications & notes** |
| --- | --- | --- | --- | --- | --- | --- | --- | --- | --- | --- | --- |
| Huurneman, B., Boonstra, F. N., & Goossens, J. (2016).  Huurneman, B., Boonstra, F. N., & Goossens, J. (2016).  Huurneman, B., Boonstra, F. N., & Goossens, J. (2017). | randomized controlled trial | 36 | 6-11 | IN (100%), IIN (50%), oculocutaneous albinism (50%) | behavioral | crowded or uncrowded computer-controlled letter-discrimination tasks (twice per w for 5 w, 3500 trials in total) | in saccade task, latencies decreased by 14 ± 4 ms and gains increased by 0.03 ± 0.01;  no training-induced changes in nystagmus characteristics and fixation stability | Method: Landolt C-chart crowded and uncrowded (distance) and LEA-version C-test (near)  Distance: 5m and 0.4m  Primary \| null: null  Distance VA:  ΔVA un: 0.10 logMAR  Δ VA cr: 0.11 logMAR  Same in both training groups.  Near VA:  ΔVA un: 0.07 logMAR  Δ VA cr: 0.15 logMAR cr training group and 0.05 logMAR in uncr training group. | reading acuity 0.12 ± 0.02 logMAR;  critical print size 0.11 ± 0.04 logMAR;  no change in acuity reserve and reading speed | posttest within 2 w after the training | 11 children with normal vision were tested for norm data and test-retest data; Age and baseline characteristics are crucial parameter responsible for the improvement in single-letter VA |
| Mohamad Fadzil, N., Mohammed, Z., Mohamad Shahimin, M., & Saliman, N. H. (2019). | randomized controlled trial | 18 | 13-18 | IN (100%) | optical + behavioral | spectacles or null zone training (for 5 w) | CHP, 2 (22%) not presented and 7 (78%) unchanged (spectacles); CHP, 2 (22%) not presented, 1 (11%) unchanged and 6 (67%) improved | Method: logMAR chart and MNread chart  Distance: distance and near (not specified)  Primary \| null: null  ΔDVA both groups n.s.  ΔNVA both groups n.s. | Reading time: spectacle group n.s., null zone group significant.  Reading rate: same as above. | N/A | N/A |
| Daibert-Nido, M., Pyatova, Y., Markowitz, M., Taheri-Shirazi, M., & Markowitz, S. N. (2021). | non-randomized intervention study | 10 | 9 ± 3.2 | IIN (100%) | behavioral | biofeedback training (20 min per w for 4 w, 80 min in total) | N/A | Method: ETDRS chart  Distance: 4m and near  Primary \| null: null  Distance VA:  Δ VA: 0.10 logMAR  Near VA:  Δ VA: 0.20 logMAR | reading speed from 74.7 ± 51.2 to 104.7 ± 53.6 words/minute (p<0.0006);  CVFQ from 23.8 ± 2.2 to 26.3 ± 2.3 (p=0.001) | posttest in 1 w; follow-up for 3 mos to 1 y | with no other ocular diseases, retinal disease, FH, both eyes with media opacity, nystagmus cases;  no side effects |
| Huurneman, B., & Goossens, J. (2021). | non-randomized intervention study | 37 | 7-18, | IN (100%), IIN (49%), oculocutaneous albinism (51%) | behavioral | prolonged home-based perceptual training (twice per w for ~20 w, 14000 trials intotal) | N/A | Method: Landolt C-chart crowded and uncrowded (distance) and LEA-version C-test (near)  Distance: 5m and 0.4m  Primary \| null: null  Distance VA:  Δ VA unc: 0.15 logMAR  Δ VA cr: 0.24 logMAR (IIN), 0.13 logMAR albinism  Near VA:  ΔVA un: 0.09 logMAR  Δ VA cr: 0.10 logMAR | Improvement in reading speed.  FVQ_CYP improvement in user ease, school activities, mobility activities but not leisure activities | posttest within 2 w; follow-up test in 6 mos after the training | 1 subject dropped out;  long-term effect of training retented;  Some patients did not reach the plateau performance. |
| Caputo, R., Febbrini del Magro, E., Amoaku, W. M., Bacci, G. M., Marziali, E., & Morales, M. U. (2021). | retrospective case series | 12 | 8.9 ± 2.07 | IN (100%), albinism (33%), hypoplasia (33%), IIN (25%), esotropia (25%), achromatopsia (8%) | behavioral | biofeedback fixation training with microperimetry (10 weekly sessions + 8 biweekly sessions + 6 monthly sessions, 10 min per session) | BCEA @95%  improved at W10 and M6 (p=0.004);  BCEA @63%  improved at W10 and M6 (p=0.01);  P1, P2 no sigificant change (p= 0.07, 0.13) | Method: not specified  Distance: not specified  Primary \| null: null  BCVA no significant change (p=0.12) | N/A | N/A | N/A |

BCVA, best corrected visual acuity; BBCVA, binocular best-corrected visual acuity; BCEA, bivariate contour ellipse area; CHP, compensatory head posture; CVFQ, Children’s Visual Function Questionnaire; DVA, distance visual acuity; FH, foveal hypoplasia; IN, infantile nystagmus; IIN, idiopathic infantile nystagmus; NAFX, eXpanded Nystagmus Acuity Function; NVA, near visual acuity; P1/P2, displacement from fixation point; QoL, quality of life; VA, visual acuity; min/w/mos/y, minute(s)/week(s)/month(s)/year(s); note: the follow-up duration signifies the time after the intervention has ceased.
